# Part-Per-Billion Level Chemical Sensing with a Gold-Based SERS-Active Substrate

**DOI:** 10.3390/s22051778

**Published:** 2022-02-24

**Authors:** Tingting Zhang, Liyun Wu, Junchang Pei, Xuefeng Li, Haowen Li, Frank Inscore

**Affiliations:** 1 Micro Optical Instruments, Shenzhen 518118, China; zttida1991@163.com (T.Z.); l.wu@moiiot.com (L.W.); h.li@moiiot.com (H.L.); 2 Institute of Criminal Science and Technology, Taizhou Public Security Bureau, Taizhou 225300, China; peijunchang123@163.com (J.P.); leexf2022@126.com (X.L.)

**Keywords:** surface-enhanced Raman sensor, SERS, trace chemical detection, Au nanoparticles

## Abstract

We used surface-enhanced Raman spectroscopy (SERS) for the rapid and sensitive detection and quantification of caffeine in solution. Such a technique incorporated into a portable device is finding wide applications in trace chemical analysis in various fields, including law enforcement, medicine, environmental monitoring, and food quality control. To realize such applications, we are currently developing portable and handheld trace chemical analyzers based on SERS, which are integrated with a sensor embedded with activated gold nanoparticles in a porous glass matrix. In this study, we used this gold SERS-active substrate to measure aqueous solutions of the drug caffeine as a test chemical to benchmark sensor performance by defining sensitivity (lowest measured concentration (LMC) and estimated limit of detection (LOD)), determining concentration dependence and quantification capabilities by constructing calibration curves; by evaluating the effects of pH values of 3, 7, and 11; and by examining the reproducibility of the SERS measurements. The results demonstrate that the SERS sensor is sensitive, with caffeine detected at an LMC of 50 parts per billion (ppb) with an LOD of 0.63 ppb. The results further show that the sensor is very stable and can be used to make reproducible measurements, even under extremely acidic to basic pH conditions. Vibrational assignments of all observed SERS peaks are made and reported for the first time for caffeine on a gold substrate.

## 1. Introduction

Surface-enhanced Raman spectroscopy (SERS) has become a very powerful optical technique and analytical tool that is finding wide use in a variety of application areas [1,2,3,4,5,6,7]. This surge in use can be attributed to the extreme high molecular specificity and sensitivity this technique affords, with single-molecule detection being possible [8,9]. Raman and SERS enhancement theories have been extensively discussed in the literature [10,11,12,13,14,15].

A variety of SERS substrates have been characterized and extensively discussed in the literature [12,16,17,18], including numerous studies and reviews on using SERS for trace chemical detection applications [16], many having utilized silver and gold colloidal solutions. However, regarding colloid substrates, there are issues with sensitivity, stability under extreme pH, and interfering spectral contributions due to the nature of the reducing agents used. In this study, a gold sol–gel substrate was investigated as a colloid alternative. Similar silver sol–gel substrates have been described in the literature [12,16]. It is well known that silver and gold spherical nanoparticles used for the purpose of SERS typically have diameters of 10–100 nm. It is also known that the absorption maximum (surface plasmon resonance) of silver nanoparticles is near 410 nm and that of gold is around 510–530 nm, and when aggregated, this plasmon band can be shifted to the NIR [19]. Previous studies reporting on a silver sol and various gold substrates show the formation of metal nanostructures of fractal aggregates [20,21,22]. Preliminary SEM data of the silver-doped sol–gel analogue of the gold sol–gel used in this study [23] produced a silica network with bound Ag nanoscale fractal aggregates (100–1000 nm diameter). The shifting of plasmon absorption afforded by the extended aggregates of Ag or Au nanoparticles embedded in the sol–gel makes this sensor useful in laser excitation from 532 to 1064 nm, and it is ideal for use with our portable 785 nm Raman system. In this study, the drug caffeine was selected as a probe molecule to evaluate this sensor, as it generates a strong SERS response at 785 nm. The SERS of caffeine has been reported for various substrates, mostly silver colloids in water, in extracts from tea and coffee, and in biofluids [24,25,26,27,28,29,30,31,32,33,34,35]. Caffeine SERS has been measured and reported at 1 ppb on silver colloids [24] and 100 ppm on gold colloids [28].

The gold SERS substrate used in this study is unique in that the gold nanoparticles are embedded in a porous sol–gel matrix, and when immobilized in a capillary or channel, the sample analyte solution is simply flowed through the reduced plug, where the target molecules dynamically and quickly come into intimate contact with the metal nanoparticles. This is not a passive process, such as applying a sample on top of a coated SERS-active surface and allowing diffusion to slowly bring the molecules into contact with the nanostructure or mixing the sample as a colloid mixture. This gold SERS-active sensor is versatile in that as little as 10 microL samples can be measured. Larger volumes can be flowed through, which will preconcentrate the target molecules on the metal surface and thereby improve sensitivity. Another advantage that this sensor offers is the sol–gel formulation, as it can be modified with different Si alkoxides, which can affect the hydrophilic and hydrophobic nature of the porous sol–gel plug and can thus fine tune the extraction capabilities and selectivity to detect a particular class of target chemicals. Due to its unique advantages, ease in manufacturing, and low cost, we are in the process of developing this sensor for a SERS-based analyzer for onsite drug detection in biofluids and other applications.

The primary aim of this paper is to benchmark and evaluate the performance of the gold SERS-active substrate to enhance the detection of caffeine as a representative drug using a portable 785 nm Raman system. This study defines the performance metrics of the sensor in terms of detection limits (experimentally observed lowest measured concentration and the estimated limit of detection), concentration dependence and quantification capabilities, stability as a function of pH, and the reproducibility of the SERS measurements.

## 2. Materials and Methods

### 2.1. Materials and Reagents

The test chemical caffeine was obtained as a certified reference material from Cerilliant via Sigma-Aldrich (Shanghai, China) at 1 mg/mL in methanol. All chemicals and reagents used to prepare and activate the gold-doped sol–gels (SERS-active capillaries) were obtained at their purest commercially available grade and used as received from Aladdin (Shanghai, China)—tetrachloroauric (III) acid trihydrate (HAuCl_4_·3H_2_O (s)), 70% nitric acid (HNO_3_ (l)), tetramethyl orthosilicate (TMOS (l)), and sodium borohydride (NaBH_4_ (s)). Methanol (l) and the pellets of KOH used to preclean and pretreat the glass capillaries, and 1N NaOH and 1N HCl used to adjust pH were obtained from Aladdin (China). The ultrapure water used to prepare aqueous solutions and make dilutions was HPLC grade and generated in-house (Millipore Milli-Q, 18.0 MΩ). All glassware and materials used to prepare samples and transfer solutions were precleaned and dried prior to use.

### 2.2. Preparation of the SERS Substrate

The gold sol–gels were prepared according to a previously published procedure [36], with modifications. Briefly, the gold chloride salt HAuCl_4_·3H_2_O was dissolved in ultrapure water, with nitric acid added as a gelation catalyst, and then mixed with an equal volume of neat TMOS. The resulting solution was thoroughly homogenized by vortexing for 1 min. The SERS capillaries (precleaned with neat MeOH and pretreated with 0.2 M KOH (aq) and then pure water) were prepared by drawing 15 microL of the gold-doped sol–gel solution into 50 mm-long, 1.5 mm-diameter borosilicate glass capillaries (cut in half from 100 mm length, Kimble (Queretaro, Mexico)) to produce 0.5 cm plugs, and then they were sealed on each end with rubber tips. The plugs were allowed to gel and cure overnight at 23.5 °C in a vacuum drying oven (DZF-6021, Yiheng Scientific Instrument Co. Ltd., Shanghai, China), after which the incorporated gold ions were reduced at room temperature with two applications of dilute aqueous NaBH_4_ and a final water wash to remove residual reducing agent. The activated SERS sensors are ready for immediate use. The non-activated sol–gel plugs tightly sealed inside the capillary remain stable and optimal for at least 30 days.

### 2.3. SERS Measurements

#### 2.3.1. Sample Preparation

Caffeine was diluted from 1 mg/mL stock (1000 parts per million or 1000 ppm) to 100 ppm using ultrapure water (Millipore Milli-Q, 18.0 MΩ) and then measured in the SERS-active capillaries. A small aliquot (~20 microL) of sample, with enough volume to cover the activated sol–gel plug, was used in each SERS measurement.

To determine the sensitivity (detection limits) of the SERS-active capillaries, expressed experimentally as the lowest measured concentration (LMC), the solution at 100 ppm was further diluted in ultrapure water to produce concentrations at 50 ppm, 10 ppm, 5 ppm, 1 ppm, 500 ppb, 100 ppb, 50 ppb, and 10 ppb (10 parts per billion or 10 ng/mL). To establish quantitation capabilities, concentration calibration curves were constructed by plotting the intensity of the baseline-corrected characteristic peak as a function of concentration. A sample at 250 ppb was used as an unknown to test quantitation.

To determine the effects of pH, samples at 1 ppm were adjusted with a minimal volume of 1N HCl or 1N NaOH to produce three solutions at a given concentration with pH = 3, 7, and 11 (PHS-3C pH meter, INESA Scientific Instrument Co. Ltd., Shanghai, China). The pH-adjusted solutions were allowed to equilibrate at room temperature prior to SERS measurements.

All measurements were performed in triplicate. A minimum of three spots were measured on each capillary. Reproducibility studies were performed on three capillaries made in one batch and a fourth capillary made in a separate batch on a different day with freshly prepared caffeine at 1 ppm in ultrapure water. Note that all gold substrate and drug sample preparations in this study were prepared in a laboratory chemical hood (Guange SFH150), and standard safety precautions were followed. The normal Raman (NR) spectrum of caffeine powder was obtained from a purchased FT-Raman library (Nicolet, Madison, WI, USA).

#### 2.3.2. Instrumentation

**Figure 1:**
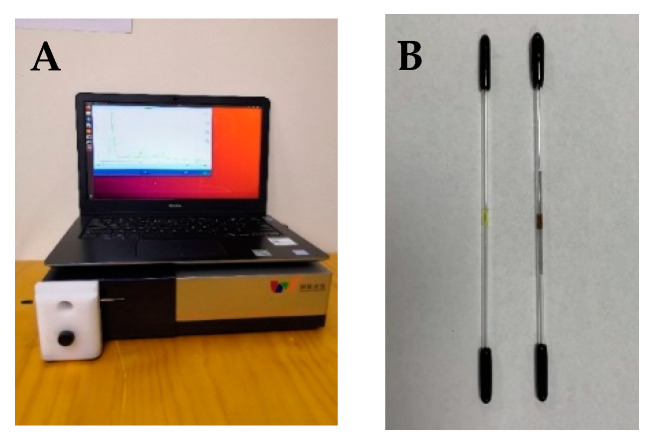
MOI instrument and sample configuration for (**A**) portable desktop Raman unit and (**B**) gold SERS capillary sensors before reduction on the left and after reduction on the right. Note that the rubber tips are attached after the sol–gel is drawn into the capillary, and they remain attached during the curing stage and in storage (on **left**) and are then removed prior to the reduction step. The reusable rubber tips are then reattached after the reducing agent is flowed through sol–gel plug and subsequent water washing step, and they remain attached until it is time to add the sample (on **right**).

Surface-enhanced Raman spectra for caffeine were measured using a desktop dispersive Raman spectrometer (MOI, DTR785-1) manufactured in-house, and it provided 100–2875 cm^−1^ spectral coverage with variable 7–10.5 cm^−1^ resolution as shown in Figure 1A. The SERS-active capillaries were fixed horizontally to an XY positioning stage (custom made at MOI). The SERS was measured with 187.5 mW (50%) of 785 nm laser excitation, with the probe configured to collect the 180 degree backscattered radiation. The integration time was set to 0.3 s, with SERS spectra obtained based on the average of 4 scanning results. The SERS spectra were preprocessed, including a baseline correction, and were clipped from 400 to 1800 cm^−1^ to capture the regions of most important spectral significance. This system is integrated into a small case and is made field portable with battery operation. Our instruments are pre-calibrated by a standard operational procedure, and a quick calibration can be automatically performed at the time the instrument is turned on with a small rod of polystyrene.

### 2.4. Data Analysis

The analysis of spectroscopic data was accomplished by the following: Spectral peak positions for the NR and SERS were determined and calculation of signal/noise were performed with an in-house LabVIEW-based program. Data processing (baseline correction), construction of concentration calibration curve, and linear regression analysis were performed with Origin 8.0. Limit of detection (LOD) was calculated by two different approaches (following ICH guidelines). (1) The first approach was based on the signal-to-noise ratio, LOD = (C)/([S/N]/3), where C is the concentration in ng/mL, S is the signal baseline-corrected peak intensity, N is the standard deviation noise, and 3 is the cutoff peak signal relative to the background or blank. (2) The second approach was based on the calibration curve, LOD = 3.3 σ/S, where σ is the standard error of the regression, and S is the slope of the linear portion of the curve. Statistical analysis of reproducibility data was performed within Microsoft Excel.

## 3. Results and Discussion

### 3.1. SERS Spectral Reference Measurement of Caffeine and Band Assignments

**Figure 2:**
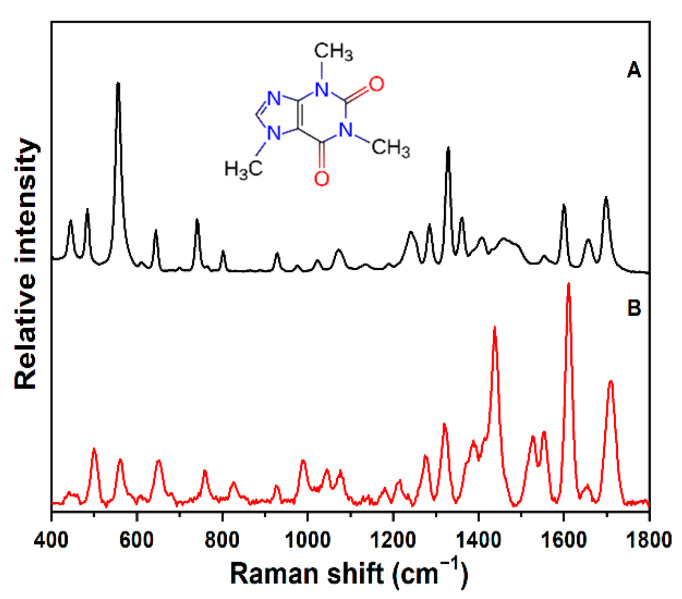
(**A**) Raman spectra of caffeine powder (with structure of caffeine shown) from commercial Nicolet spectral library, FT-Raman, 500 mW at 1064 nm with 4 cm^−1^ resolution. (**B**) SERS of caffeine, measured at 100 ppm in ultrapure water, in SERS-active gold sol–gel capillaries, on portable desktop dispersive Raman unit, 187.5 mW at 785 nm laser excitation, acquisition 300 ms with 4× averaging. The spectra were normalized to account for the different responses between the two Raman instruments (where the intensities of the dominant Raman peak at 556 cm^−1^ and the SERS peak at 1609 cm^−1^ were set to 1.0) and then offset for clarity.

The normal Raman (NR) and SERS of caffeine were measured and analyzed as described in the Materials and Methods Section, and they are presented in Figure 2 for comparison. Table 1 summarizes the peak positions, shifts, and relative intensities of the bands observed in the NR and SERS. The vibrational assignments of each observed Raman feature are based on a combination of experimental and computational results previously reported for the NR of solid and solution caffeine [32,37,38,39,40]. An examination of the NR spectrum in Figure 2A shows two intense prominent features at 556 cm^−1^, which can be assigned to an O=C-N deformation (bending mode) or, alternatively, to a pyridine ring breathing mode, and at 1329 cm^−1^, which can be assigned as an imidazole trigonal ring stretch, both characteristic of caffeine Raman. Two additional strong features characteristic of caffeine Raman are observed at 1600 cm^−1^ and at 1698 cm^−1^, which can be assigned as symmetric C=C and in-plane C=O stretches, respectively. The out-of-plane C=O stretch counterpart is attributed to the peak of weaker intensity at 1657 cm^−1^. These and the other reported assignments for caffeine NR (Table 1) form the basis for assigning the SERS features observed on our gold sensor.

Initially, the SERS of caffeine was collected at a nominal concentration of 100 ppm in ultrapure water using our activated gold sol–gel immobilized in glass capillaries, and it is shown in Figure 2B. A comparison of the SERS to the NR shows spectral differences. This is not unexpected, as the observed differences in peak position and the relative intensity reflect variations in the surface interactions between the caffeine molecules and gold nanoparticles, which enhance and shift various vibrational modes to different degrees. It is worth pointing out that vibrational assignments of caffeine SERS on various silver substrates have been made in reference to caffeine NR features [24,32], but, to the best of our knowledge, similar assignments for all observed SERS bands have not been reported on gold. Furthermore, the SERS spectral features presented here for gold are quite similar to the SERS of caffeine on silver reported at basic pH, in which density functional calculations were used to help make corresponding assignments in the NR and SERS [32].

Based on the previous band assignments of the NR and silver SERS at basic pH, and the observed peak positions and relative intensities summarized in Table 1, we made similar corresponding assignments for the SERS bands of caffeine on gold. We assigned the dominant SERS peak at 1610 cm^−1^ and the strong peaks at 1437 cm^−1^ and 1707 cm^−1^ as a C=C stretch, imidazole ring stretch, and i.p. C=O stretching mode to the corresponding peaks assigned in the Raman spectrum, respectively. Additional unique peaks observed as doublets at 1277 and 1314 cm^−1^ and 1522 and 1551 cm^−1^, with three distinct peaks found at 499, 557 and at 650 cm^−1^, are assigned in Table 1. We made further band assignments of the observed SERS spectral features on the gold substrate to those corresponding features in the NR, and these are tentatively assigned in Table 1. The most obvious contrast observed between the NR and SERS is that the dominant NR peak at 556 cm^−1^ (O=C-N deformation) is weakly enhanced and only shifted to 557 cm^−1^ in the SERS, while modes involving the imidazole ring and C=C and C=O stretches at 1458, 1600, and 1698 cm^−1^, respectively, are greatly enhanced and significantly shifted in the SERS, indicative of a strong interaction with the gold surface.

It is worth noting that the absorption spectrum of caffeine in water reveals a band maximum at 273 nm with zero intensity at wavelengths above 302 nm [41], which precludes any direct resonance enhancement contribution to the spectrum with 785 nm laser excitation as used here in this study. The relative intensity enhancement patterns and magnitude of the peak shifts summarized in Table 1 suggest that both electromagnetic and charge transfer mechanisms contribute to the overall SERS spectra of caffeine on this gold substrate, as has been suggested for caffeine on silver.

One last point to make here is that the observed SERS features are consistent with the reported spectra for caffeine on gold colloid substrates [27,28]. However, it is important to note that the SERS spectra presented in this study are not complicated by additional interfering peaks as has been observed in previous papers, which attributed such contributions to the reducing and capping agents used in the synthesis of the gold and silver colloid solutions to make measurements of caffeine and other drugs. This absence of interfering contributions in the spectral regions of significance is an important feature of this gold substrate and allows for a more effective SERS library to be constructed for chemical identification purposes.

### 3.2. SERS LMC Determination, LOD Estimation, and Quantification of Caffeine

**Figure 3:**
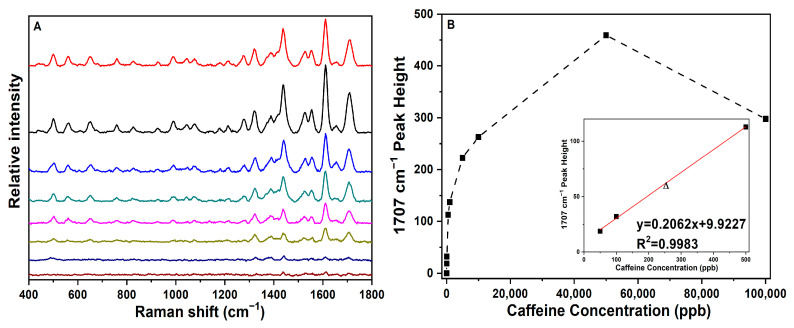
(**A**) SERS stacked plot of caffeine at 100 ppm, 50 ppm, 10 ppm, 5 ppm, 1 ppm, 500 ppb, 100 ppb, and 50 ppb (top to bottom) in water on SERS-active gold sol–gel capillaries; all SERS spectral conditions are the same as those in Figure 2. (**B**) Plot of 1707 cm^−1^ peak height as a function of caffeine concentration corresponding to the spectra in (**A**) (solid squares connected by dashed line for clarity over the entire concentration range measured). The inset shows the linear fit used in the low-concentration regime from the LMC at 50 ppb to the plateau point at 500 ppb. Triangle represents a 250 ppb caffeine test sample as an unknown.

The sensitivity of the gold SERS-active substrate was examined for caffeine. Samples in ultrapure water were measured by SERS across a range of different concentrations as mentioned in the Materials and Methods Section. The sensitivity (limit of detection) is experimentally defined here as the lowest measured concentration (LMC) in which the characteristic and unique SERS spectral peaks can still be observed in the solution matrix. The LMC spectra and corresponding concentration data are presented below in Figure 3. The LMC for caffeine was found to be 50 ppb (50 ng/mL).

The SERS (stacked plot) in Figure 3A reveals that the spectral peaks are preserved over the concentration range used to determine the LMC. As the concentration was lowered, the observed spectra were in general similar with respect to peak positions and relative intensities. This stability of peak positions at lower concentrations is important, as it allows for the detection and identification of trace amounts of drugs in unknown samples.

The LMC spectra of caffeine were used to calculate a theoretical limit of detection (LOD) based on a signal-to-noise ratio (S/N) of three (that is, by assigning a cut-off peak signal as three times the background signal). The baseline-corrected height of the peak being profiled was divided by the standard deviation noise for the spectral interval between 40 and 60 cm^−1^, where there is only detector noise. The estimated LOD for caffeine using the unique characteristic peak at 1707 cm^−1^ was calculated as follows: LOD = (LMC)/([Signal/std Noise]/3) = (50 ng/mL)/([18.8/0.52]/3) = 4.1 ng/mL or 4.1 parts per billion.

Figure 3B shows the concentration calibration curve constructed for caffeine. The baseline-corrected intensity of the strong unique characteristic peak at 1707 cm^−1^ was plotted as a function of the sample concentration. The SERS peak intensities measured over the entire concentration range were found to exhibit a nonlinear relationship that follows a standard Langmuir adsorption isotherm curve [42]. For caffeine, the intensity begins to plateau above 500 ppb and is observed to rollover above 50 ppm such that the intensity at 100 ppm is less than that at 50 ppm. This plateauing effect and rollover are attributed to the higher drug concentration saturating the surface of the gold nanoparticles (i.e., as increasingly more drug molecules adsorb to the surface and exceed monolayer coverage, the signal will level and begin to diminish). However, the region between 50 ppb and 500 ppb is linear and can be used for quantitative purposes. This linear region for peak intensity versus concentration is presented in the inset in Figure 3B, along with the correlation coefficient R^2^ of the best fit equation, y = mx + b. The quantitation aspect of the concentration curve was tested. As shown in Figure 3B, the triangle represents a 250 ppb caffeine test sample (as an unknown) and is plotted against the best fit line. Based on the 1707 cm^−1^ peak height, the linear equation predicted the concentration to be X = [(58 − 9.92)/0.206] = 233.3 ppb, where the % error |(233.3 − 250)/250|(100) = 6.7%. This demonstrates that such drugs in unknown samples can potentially be quantitatively detected at ppb levels with this gold SERS sensor. The LOD can also be calculated from the regression analytics obtained from the linear portion of the calibration curve, and it is more robust than the S/N approach above. Here, LOD = 3.3 σ/S, where σ is the standard error of the regression of the slope, and S is the slope of the linear portion of the curve and gives LOD = (3.3 × 0.0248)/0.137) = 0.63 ng/mL (0.63 ppb), which is lower than that calculated above by the S/N approach (4.1 ppb).

### 3.3. Effects of pH on Caffeine Measurements with the SERS-Active Substrate

**Figure 4:**
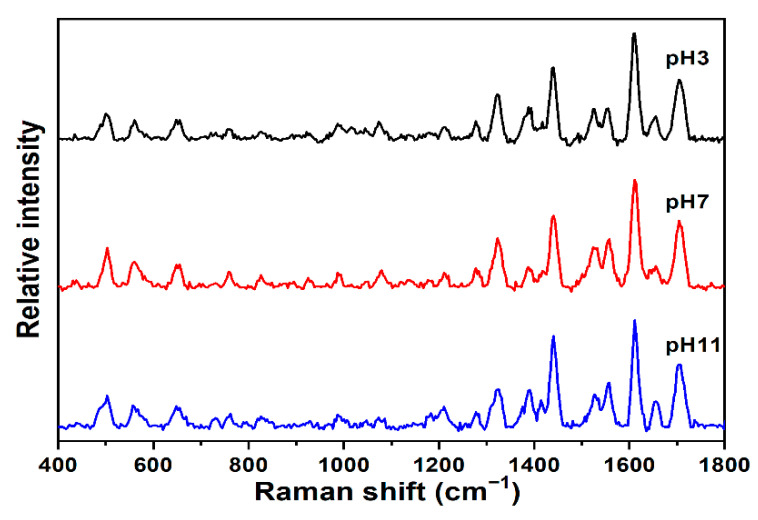
SERS of 1 ppm caffeine in water on SERS-active gold sol–gel capillaries at pH = 3, 7, and 11; all other spectral conditions for SERS are the same as those in Figure 2. Note, caffeine pKa = 10.4.

The possibility of pH effects on the SERS measurements was examined for caffeine on the SERS-active gold substrate. Previously, pH-dependent SERS effects have been reported for caffeine on silver colloids [32]. As described in the Materials and Methods Section, aqueous solutions of caffeine were prepared and pH adjusted to values of 3, 7, and 11. The results (SERS spectra, stacked plot at pH = 3, 7, and 11) are presented in Figure 4. The spectra are very similar and exhibit no significant changes, as the pH is varied with respect to the peak position and relative peak intensity on the SERS-active gold substrate used here. A previous study on caffeine also noted that the SERS at pH > 10 and pH < 2 was weak due to the instability of silver colloids [24]. However, as seen in Figure 4, the signal of caffeine is clearly stable on this gold SERS-active substrate at both extreme acidic and basic pH. The study on silver colloids shows the acidic spectra and basic spectra to be different due to the orientation and form of the caffeine adsorbed onto the surface. It is clear in Figure 4 that caffeine orients and adsorbs the same, from acidic to basic pH, on the surface of this gold sensor.

### 3.4. Reproducibility of the SERS Substrate with Caffeine

Table 1 below presents the reproducibility data for 1 ppm caffeine measured on 3 gold SERS-active capillaries prepared in the same batch at 10 different spots on each using our portable desktop Raman unit. Five spots were measured at 1 mm intervals along the 0.5 cm sol–gel plug, and five additional spots were measured by rotating the capillary. A fourth gold SERS-active capillary prepared at a different time (from a different batch) was also measured for comparison. The 1707 cm^−1^ peak height measured on each spot for each capillary was baseline corrected, and the statistical analysis of this peak is tabulated in Table 2. The % deviation between the three capillaries of batch1 measured for 1 ppm caffeine on our portable desktop unit is 14.30%, and the % deviation between all four individual capillaries of both batches is 15.43%, with the average % deviation between the two batches being 16.56%. These results demonstrate that reliable measurements can be made on the gold SERS sensors within a batch and from batch-to-batch preparations. Standardization and automation of the entire process to manufacture these sensors should greatly improve the overall reproducibility of the SERS measurements.

## 4. Conclusions

A modified sol–gel method incorporating gold nanoparticles was utilized to make a universal sensor to detect trace chemicals in solution, in this case, the naturally occurring drug caffeine. The LMC of caffeine was experimentally observed at 50 ng/mL (50 ppb) with an LOD calculated at 0.63 ng/mL (0.63 ppb) on our portable desktop unit, which is the lowest detection limit yet reported for this drug on gold. Importantly, the results clearly show that the gold SERS-active sensors in this study are very sensitive, quantitative, and reproducible, and they are also very stable and can make reliable measurements over the pH range under extreme acidic to extreme basic conditions. This sensor is currently being developed in our lab for future applications involving biomarker and drug analyses in biofluids.

## Figures and Tables

**Table 1:** The observed peaks in the Raman and SERS spectra and their vibrational assignments. Raman bands were assigned according to the literature, and SERS bands were tentatively assigned to corresponding NR features.

Raman (cm^−1^)	SERS (cm^−1^)	Δ (cm^−1^)	Vibrational Assignments
445 s	442 w	−3	N-C-C deformation
483 s	499 m	+16	C-N-C deformation
556 vs	557 m	+1	O=C-N deformation (or pyridine ring breathing mode)
610 vw	610 vw	0	o.p. CH deformation
644 s	650 m	+6	O=C-N deformation
699 vw	679 vw sh	−20	pyrimidine, imidazole ring deformation
741 s	758 m	+17	O=C-C deformation
764 vw sh	-		
802 m	822 w	+20	N-C-H deformation
928 m	925 w	−3	imidazole ring deformation
975 w	987 m	+12	pyrimidine ring deformation
1023 w	1044 m	+21	i.p. C-C deformation
1072 m	1076 m	+4	H-C=N bending
1135 w	1138 vw	+3	CH3 bending
1190 vw	1179 w	−11	CH bending
1241 s	1217 w	−24	C-N stretching
1285 s	1278 m	−7	C-N stretching
1329 vs	1319 s	−10	imidazole trigonal ring stretching
1361 s	1369 m sh	+8	C=N, C-N stretching
1384 w sh	1387 s sh	+3	CH2 bending
1408 m	1411 m sh	+3	C-N sym. stretching
1458 m	1437 vs	−21	CH2 bending + imidazole ring stretching
1500 m sh	1526 s	+26	CHn bending + C-N stretching
1554 w	1552 s	−2	imidazole, pyrimidine ring stretching
1600 s	1610 vs	+10	C=C sym. stretching
1657 m	1655 w	−2	o.p. C=O stretching
1698 s	1707 vs	+9	i.p C=O stretching

vs = very strong, s = strong, m = medium, w = weak, vw = very weak, sh = shoulder, i.p. = in plane, o.p. = out of plane.

**Table 2:** Peak heights and statistics for SERS measurements of 1 ppm caffeine in water in four gold SERS-active capillaries prepared in batches (spectral conditions the same as those in Figure 2).

**1 ppm**	**Batch1**			**Batch2**	Average
	**cap1**	**cap2**	**cap3**	**cap4**	
spot1	118	160	125	152	
spot2	116	174	179	170	
spot3	142	160	159	164	
spot4	143	111	170	167	
spot5	148	116	184	174	
spot6	167	152	176	118	
spot7	132	156	163	117	
spot8	105	158	163	109	
spot9	125	163	135	134	
spot10	122	162	114	110	
Avg	131.8	151.2	156.8	141.5	145.325
Stddev	18.4	20.7	24.0	26.7	22.4
%Dev	13.93%	13.69%	15.29%	18.83%	15.43%

## Data Availability

All data used to support the findings of this study are included in the article.
